# Children's body mass index, participation in school meals, and observed energy intake at school meals

**DOI:** 10.1186/1479-5868-7-24

**Published:** 2010-03-24

**Authors:** Suzanne Domel Baxter, James W Hardin, Caroline H Guinn, Julie A Royer, Alyssa J Mackelprang, Christina M Devlin

**Affiliations:** 1 Institute for Families in Society, University of South Carolina, 1600 Hampton Street, Suite 507, Columbia, SC 29208 USA; 2 Department of Epidemiology and Biostatistics, University of South Carolina, Columbia, SC 29208 USA

## Abstract

**Background:**

Data from a dietary-reporting validation study with fourth-grade children were analyzed to investigate a possible relationship of body mass index (BMI) with daily participation in school meals and observed energy intake at school meals, and whether the relationships differed by breakfast location (classroom; cafeteria).

**Methods:**

Data were collected in 17, 17, and 8 schools during three school years. For the three years, six, six, and seven of the schools had breakfast in the classroom; all other schools had breakfast in the cafeteria. Information about 180 days of school breakfast and school lunch participation during fourth grade for each of 1,571 children (90% Black; 53% girls) was available in electronic administrative records from the school district. Children were weighed and measured, and BMI was calculated. Each of a subset of 465 children (95% Black; 49% girls) was observed eating school breakfast and school lunch on the same day. Mixed-effects regression was conducted with BMI as the dependent variable and school as the random effect; independent variables were breakfast participation, lunch participation, combined participation (breakfast and lunch on the same day), average observed energy intake for breakfast, average observed energy intake for lunch, sex, age, breakfast location, and school year. Analyses were repeated for BMI category (underweight/healthy weight; overweight; obese; severely obese) using pooled ordered logistic regression models that excluded sex and age.

**Results:**

Breakfast participation, lunch participation, and combined participation were not significantly associated with BMI or BMI category irrespective of whether the model included observed energy intake at school meals. Observed energy intake at school meals was significantly and positively associated with BMI and BMI category. For the total sample and subset, breakfast location was significantly associated with BMI; average BMI was larger for children with breakfast in the classroom than in the cafeteria. Significantly more kilocalories were observed eaten at breakfast in the classroom than in the cafeteria.

**Conclusions:**

For fourth-grade children, results provide evidence of a positive relationship between BMI and observed energy intake at school meals, and between BMI and school breakfast in the classroom; however, BMI and participation in school meals were not significantly associated.

## Background

It is well known that the incidence of obesity has increased dramatically over the last several decades among children in the United States [1,2] and around the world [3]. This is of concern because overweight children are at increased risk for morbidity and mortality later in life [4].

The School Breakfast Program (SBP) and the National School Lunch Program (NSLP) are two food assistance programs administered by the United States Department of Agriculture. Millions of children participate in the SBP and NSLP each school day [5]. Children from families with incomes ≤ 130% of the poverty level are eligible for free meals, and children from families with incomes between 130% and 185% of the poverty level are eligible for reduced-price meals [6].

There is growing concern about whether participation in school meal programs contributes to obesity [7-9]. In a review of eating patterns and obesity, Nicklas and colleagues [10] commented that although the SBP and NSLP have had a major, generally positive impact on children's nutritional status, "*the introduction of school lunch and later school breakfast has likely contributed to obesity in low-socioeconomic-status children*." An Expert Panel was convened in March, 2004, by the United States Department of Agriculture to identify evidence about whether participation in food assistance programs contributes to obesity [9]. The Panel concluded that *"the sparse research that has been published provides no consistent evidence of association" *between participation in either the SBP or the NSLP and overweight or obesity [9].

To our knowledge, two feeding trials have investigated a relationship between participation in school meal programs and obesity. Paige [11] conducted a one-year lunch program at four elementary schools; results failed to indicate significant differences in mean increases in weight or height for fed and non-fed lunch groups after one school year. Lieberman and colleagues [12] offered free breakfast at a test elementary school but not at a control school; repeat anthropometric measurements at the test school after five months of free breakfast revealed no significant shift from initial measurements for any age or sex group.

We are aware of six non-feeding studies that have investigated a relationship between participation in school meal programs and obesity. Anthropometric results from the National Evaluation of School Nutrition Programs indicated that SBP participation was not related to height and only weakly and positively related to weight and triceps fatfold; also, NSLP participation was not related to height but was weakly and positively related to weight, which was at least partly due to an increase in triceps fatfold [13]. Wolfe and colleagues [14] found that elementary school children who tended to be fatter ate school lunch. Melnik and colleagues [15] found that overweight among elementary school children was not associated with NSLP participation. Jones and colleagues [16] found that for food-insecure children, SBP and NSLP participation was inversely associated with risk of overweight; however, for food-secure children, there was no difference in the risk of overweight by program participation. Hofferth and Curtain [17] commented that SBP and NSLP participation "*may be associated with a higher chance of being overweight...children who ate a school lunch had a significantly higher BMI and a greater chance of being overweight than those who did not, and those who ate a lunch and breakfast also had a significantly higher BMI and chance of being overweight*." For the third School Nutrition Dietary Assessment Study, children reported the number of times per week (zero to five) that they a) "usually eat a school breakfast" and b) "usually eat a school lunch"; although results showed no relationship between participation in the NSLP and body mass index (BMI) percentile, a negative relationship was found between participation in the SBP and BMI percentile [18].

In previous research concerning a possible relationship between childhood obesity and SBP and NSLP participation, two methodological limitations are evident. First, information about SBP and NSLP participation has relied on parental yes/no responses, or children's reports about the past few days or usual times per week. Such proxy- and self-reports are prone to error [19]. Second, information about children's intake at school meals has relied on parental and/or children's reports. However, results from validation studies indicate that parents have difficulty accurately reporting their children's intake [20-22] and that children's dietary reporting errors can be substantial [23-26]. To our knowledge, no previous research concerning a possible relationship between childhood obesity and SBP and NSLP participation has utilized daily SBP and NSLP participation, or observed intake at school meals.

When investigating a possible relationship between childhood obesity and SBP and NSLP participation, it may be important to consider the location of school breakfast. The *School Breakfast Program Pilot Projec*t [27] studied the impact of universal-free school breakfast on SBP participation and other measures. Results indicated that SBP participation was significantly higher for breakfast in the classroom (66%) than in the cafeteria (28%) [27]. Also, two or more breakfasts (at home and at school) on a given day were reported as eaten for a larger share of children with breakfast in the classroom than children without breakfast in the classroom, and higher intakes of energy at breakfast (but not over 24 hours) were reported for children with breakfast in the classroom than children without breakfast in the classroom [27]. (For children without breakfast in the classroom, the school cafeteria was the primary location for school breakfast.) No significant differences were found in children's BMI percentile between schools with universal-free school breakfast and schools with regular SBP (i.e., without universal-free school breakfast) [28]. However, *daily *SBP participation was *not *investigated, and children's energy intake was not observed but instead was reported by children and/or their parents.

For this article, data collected during three school years for a dietary-reporting validation study with fourth-grade children [29] were analyzed to address three research questions (RQ):

### RQ1

What is the relationship between children's BMI and daily school meal participation during the fourth-grade school year? Does this differ for breakfast and lunch participation separately versus combined, by sex, by age (in months), by breakfast location (cafeteria; classroom), and by school year?

### RQ2

For a subset of fourth-grade children observed eating school meals, what is the relationship between BMI and observed energy intake at school meals? Does this differ for breakfast and lunch separately versus combined, by sex, by age, by breakfast location, and by school year?

### RQ3

For a subset of fourth-grade children observed eating school meals, what is the effect on RQ1 when observed energy intake at school meals is included in the analyses?

## Methods

The institutional review board at the University of South Carolina approved the project. Written parental consent and child assent were obtained. Data collection methods for the dietary-reporting validation study have been described in detail elsewhere [29]; thus, only a summary is provided here.

Data were collected in one district during three school years. During the 2004-05 school year, 17 schools participated. During the 2005-06 school year, 16 of the same schools participated along with an additional school, for a total of 17 schools. During the 2006-07 school year (when data collection needs were less), eight schools participated that had each participated in both of the first two school years. Breakfast location was not manipulated for the dietary-reporting validation study, but was determined by each school principal and was unchanged during a given school year. For the three respective school years, six, six, and seven schools had breakfast in the classroom; all other schools had breakfast in the cafeteria. Across the three school years of data collection, only one school changed its breakfast location; that school had breakfast in the cafeteria for the first two school years, and breakfast in the classroom for the third school year. The district had implemented offer-vs-serve foodservice, so children could refuse some meal items [30]. For the respective three school years, of the 933, 959, and 499 fourth-grade children invited to participate, 687 (74%), 733 (76%), and 360 (72%) agreed, for an overall total of 1,780 children in the study.

### Participation in school meals

Information about 180 possible days of school breakfast and school lunch participation during children's fourth-grade school year was obtained from electronic administrative records provided by the school district. This information was available for 1,571 (90% Black; 53% girls) of the 1,780 fourth-grade children in the study. For this article, breakfast participation was defined as the number of days on which a child participated in school breakfast during his or her fourth-grade school year. Lunch participation was defined as the number of days on which a child participated in school lunch during his or her fourth-grade school year. Combined participation was defined as the number of days on which a child participated in both school breakfast and school lunch during his or her fourth-grade school year.

### Weight/height measurements and age

Research staff followed a written protocol and standardized procedures [31,32] to weigh and measure children in the morning (after school breakfast but before school lunch) in the spring of their fourth-grade school year using digital scales and portable stadiometers. Inter-measurer reliability was assessed daily for pairs of research staff on measurements from a random 10% sample of children. Intraclass correlation reliability was > 0.99 for weight and for height for each of the three school years. Each child's age was calculated by subtracting his or her date of birth (obtained from school records) from the date of measurement. Each child's BMI percentile was determined based on age/sex BMI charts [33,34]. Each child was assigned to a BMI category based on the recommendations of the Expert Committee for the Prevention, Assessment, and Treatment of Child and Adolescent Overweight and Obesity [35]. These BMI categories consisted of *underweight *(< 5^th ^percentile), *healthy weight *(≥ 5^th ^to < 85^th ^percentiles), *overweight *(≥ 85^th ^to < 95^th ^percentiles), *obese *(≥ 95^th ^to < 99^th ^percentiles), and *severely obese *(≥ 99^th ^percentile).

### Observed intake at school meals

Each of a subset of 465 fourth-grade children (95% Black; 49% girls) was observed eating school breakfast and school lunch on the same day during data collection. Of the 465 children, both school meals were observed eaten on one day for 381 children, on two days for 73 children, on three days for 10 children, and on four days for 1 child, for a total of 561 days of observed school meal intake across the 465 children. For observations of school meals, researchers observed one to three children simultaneously during regular meal periods with children seated according to their school's typical arrangement. Entire meal periods were observed so that food trades (items received and/or given away) could be noted. Researchers used paper forms to record items and amounts eaten in servings of standardized school meal portions. Interobserver reliability [36], which was assessed weekly, was acceptable [29].

Amounts eaten of each serving were recorded as none, taste, little bit, half, most, all, or as the actual number of servings if more than one serving was observed eaten. Qualitative labels used to record observed information were assigned numeric values as none = 0.0, taste = 0.1, little bit = 0.25, half = 0.5, most = 0.75, all = 1.0, or as the actual number of servings if more than one serving was observed eaten. Quantified observed servings were multiplied by per-serving energy values (in kilocalories) of standardized school meal portions. These energy values were obtained primarily from the Nutrition Data System for Research (Nutrition Coordinating Center, University of Minnesota, Minneapolis, MN); however, sometimes the school district's nutrition program provided this information. For each observed meal, the values for energy intake were summed across items eaten. For each child with multiple days of observations, average observed energy intakes for breakfast and for lunch were calculated.

### Analyses for RQ1

Mixed-effects regression was conducted with BMI as the dependent variable and school as the random effect. The independent variables were breakfast participation, lunch participation, combined participation, sex, age (in months), breakfast location (classroom; cafeteria), and school year. The sample for RQ1 was comprised of 1,571 children.

### Analyses for RQ2

Mixed-effects regression was conducted with BMI as the dependent variable and school as the random effect. The independent variables were average observed energy intake for breakfast (in units of 100 kilocalories), average observed energy intake for lunch (in units of 100 kilocalories), sex, age, breakfast location, and school year. The sample for RQ2 was comprised of 465 children.

### Analyses for RQ3

Mixed-effects regression was conducted with BMI as the dependent variable and school as the random effect. The independent variables were breakfast participation, lunch participation, combined participation, average observed energy intake for breakfast (in units of 100 kilocalories), average observed energy intake for lunch (in units of 100 kilocalories), sex, age, breakfast location, and school year. The sample for RQ3 was comprised of 465 children.

### Analyses for all three RQs

In the analyses just described for each RQ, BMI was used instead of BMI percentile because numerous BMI percentile values were near the upper bound. For each of the three RQs, analyses were repeated for BMI category using pooled ordered logistic regression models; the modified sandwich variance estimator [37] adjusted standard errors for multiple observations for schools. Because BMI percentiles are based on age and sex, those measures were not included as covariates in models for BMI category. Instead of using five BMI categories for these analyses, four BMI categories were used; the very few (20 of 1,571) children in the *underweight *(< 5^th ^percentile) BMI category were included in the *healthy weight *(≥ 5^th ^to < 85^th ^percentiles) BMI category.

### Post-hoc analyses

Post-hoc two sample *t*-tests investigated differences in observed energy intake for breakfast and for lunch by breakfast location. Additional post-hoc two sample *t*-tests investigated differences in observed energy intake for breakfast and for lunch by sex. The sample for post-hoc analyses was comprised of 465 children.

## Results

Table 1 shows the number and percent of children by BMI category and school year. This information is shown for the 1,571 children for whom participation information was available, as well as for the subset of 465 children for whom observed intake at school meals was available.

**Table 1:** Information about children by BMI category and school year for the total sample and subset^a^

	2004-05 school year		2005-06 school year		2006-07 school year	
	
	All children n = 616	Subset n = 187	All children n = 646	Subset n = 213	All children n = 309	Subset n = 65
	
BMI category	- - - - - - - - - - - - - - - - - - - - - - - - n (%) - - - - - - - - - - - - - - - - - - - - - - - - -					
Underweight & healthy weight (< 85^th ^percentile)	322 (52%)	103 (55%)	347 (54%)	102 (48%)	142 (46%)	27 (42%)

Overweight (≥ 85^th ^to < 95^th ^percentiles)	116 (19%)	29 (16%)	123 (19%)	44 (21%)	56 (18%)	14 (22%)

Obese (≥ 95^th ^to < 99^th ^percentiles)	139 (23%)	41 (22%)	112 (17%)	43 (20%)	70 (23%)	16 (25%)

Severely obese (≥ 99^th ^percentile)	39 (6%)	14 (8%)	64 (10%)	24 (11%)	41 (13%)	8 (12%)

^a^Each child in the subset was observed eating school breakfast and school lunch (with both meals observed on the same day).

Table 2 provides means and standard deviations for the number of days of breakfast participation, lunch participation, and combined participation, by BMI category for all 1,571 children, and for the subset of 465 observed children.

**Table 2:** School meal participation (in days) by BMI category for the total sample and subset^a^

	Underweight & healthy weight (< 85^th ^percentile)		Overweight (≥ 85^th ^to < 95^th ^percentiles)		Obese (≥ 95^th ^to < 99^th ^percentiles)		Severely obese (≥ 99^th ^percentile)	
	
	All children n = 811	Subset n = 232	All children n = 295	Subset n = 87	All children n = 321	Subset n = 100	All children n = 144	Subset n = 46
	
Participation	**- - - - - - - - - - - - - - - - - - - - - - - - - - - - mean (standard deviation) - - - - - - - - - - - - - - - - - - - - - - - - - - - - - - - - - -** - - - - -							
Breakfast^b^	104 (58)	141 (27)	104 (57)	141 (25)	106 (58)	142 (25)	107 (55)	140 (24)

Lunch^c^	156 (28)	162 (16)	155 (32)	163 (11)	156 (29)	161 (14)	157 (25)	163 (10)

Combined^d^	98 (56)	133 (26)	97 (55)	133 (25)	99 (55)	133 (26)	100 (53)	133 (23)

^a ^Each child in the subset was observed eating school breakfast and school lunch (with both meals observed on the same day).

^b ^Breakfast participation was the number of days on which a child participated in school breakfast during his or her fourth-grade school year.

^c ^Lunch participation was the number of days on which a child participated in school lunch during his or her fourth-grade school year.

^d ^Combined participation was the number of days on which a child participated in both school breakfast and school lunch during his or her fourth-grade school year.

Children were in the fourth grade and had a mean age of 122.88 months. However, age ranged from 90 to 149 months.

### RQ1

For analysis of BMI, sex was significant (p < 0.001; coefficient for boys = —0.94); average BMI was 0.94 kg/m^2 ^smaller for boys (20.56) than girls (21.44). For the healthy weight, overweight, obese, and severely obese BMI categories, 51%, 43%, 44%, and 45% were boys, respectively.

For analysis of BMI, age was significant (p = 0.006; coefficient = 0.06); BMI increased by 0.06 kg/m^2 ^as age increased by one month.

For analysis of BMI, breakfast location was significant (p = 0.012; coefficient for classroom = 0.88); average BMI was larger for children with breakfast in the classroom (21.50) than in the cafeteria (20.54). However, for analysis of BMI category, breakfast location was not significant (p = 0.054; odds ratio for classroom = 1.31); for the healthy weight, overweight, obese, and severely obese BMI categories, 48%, 50%, 53%, and 63% had breakfast in the classroom, respectively.

Breakfast participation, lunch participation, and combined participation were not significant for analysis of BMI or BMI category (all p values > 0.602). Also, school year was not significant for analysis of BMI or BMI category (all p values > 0.082).

### RQ2

For analysis of BMI, average observed energy intake for breakfast was not significant (p = 0.062; coefficient = 0.39). However, for analysis of BMI category, average observed energy intake for breakfast was significant (p = 0.040; odds ratio = 1.22); for the healthy weight, overweight, obese, and severely obese BMI categories, average observed energy intake for breakfast was 248, 261, 302, and 281 kilocalories, respectively. Thus, a child was 1.22 times as likely to be in the next heavier BMI category for every increase of 100 kilocalories in observed energy intake for breakfast.

For analysis of BMI, average observed energy intake for lunch was significant (p < 0.001; coefficient = 0.62). Thus, for every increase of 100 kilocalories in observed energy intake for lunch, there was a 0.62 kg/m^2 ^increase in BMI. Also, for analysis of BMI category, average observed energy intake for lunch was significant (p < 0.001; odds ratio = 1.21); for the healthy weight, overweight, obese, and severely obese BMI categories, average observed energy intake for lunch was 470, 514, 580, and 588 kilocalories, respectively. Thus, a child was 1.21 times as likely to be in the next heavier BMI category for every increase of 100 kilocalories in observed energy intake for lunch.

For analysis of BMI, breakfast location was significant (p = 0.021; coefficient for classroom = 1.15); average BMI was larger for children with breakfast in the classroom (21.90) than in the cafeteria (20.48). However, for analysis of BMI category, breakfast location was not significant (p = 0.090; odds ratio for classroom = 1.44).

Sex, age, and school year were not significant for analysis of BMI (all p values > 0.161). Also, school year was not significant for analysis of BMI category (all p values > 0.065).

### RQ3

For analysis of BMI, average observed energy intake for breakfast was significant (p = 0.045; coefficient = 0.42). Thus, for every increase of 100 kilocalories in observed energy intake for breakfast, there was a 0.42 kg/m^2 ^increase in BMI. Also, for analysis of BMI category, average observed energy intake for breakfast was significant (p = 0.037; odds ratio = 1.24); for the healthy weight, overweight, obese, and severely obese BMI categories, average observed energy intake for breakfast was 248, 261, 302, and 281 kilocalories, respectively. Thus, a child was 1.24 times as likely to be in the next heavier BMI category for every increase of 100 kilocalories in observed energy intake for breakfast.

For analysis of BMI, average observed energy intake for lunch was significant (p < 0.001; coefficient = 0.62). Thus, for every increase of 100 kilocalories in observed energy intake for lunch, there was a 0.62 kg/m^2 ^increase in BMI. Also, for analysis of BMI category, average observed energy intake for lunch was significant (p < 0.001; odds ratio = 1.22); for the healthy weight, overweight, obese, and severely obese BMI categories, average observed energy intake for lunch was 470, 514, 580, and 588 kilocalories, respectively. Thus, a child was 1.22 times as likely to be in the next heavier BMI category for every increase of 100 kilocalories in observed energy intake for lunch.

For analysis of BMI, breakfast location was significant (p = 0.012; coefficient for classroom = 1.29); average BMI was larger for children with breakfast in the classroom (21.90) than in the cafeteria (20.48). Also, for analysis of BMI category, breakfast location was significant (p = 0.041; odds ratio for classroom = 1.52); for the healthy weight, overweight, obese, and severely obese BMI categories, 54%, 63%, 63%, and 74% had breakfast in the classroom, respectively.

Breakfast participation, lunch participation, and combined participation were not significant for analysis of BMI or BMI category (all p values > 0.072). Sex and age were not significant for analysis of BMI (both p values > 0.149). School year was not significant for analysis of BMI or BMI category (all p values > 0.108).

### Results from post-hoc analyses

A post-hoc two sample *t*-test of observed energy intake for breakfast by breakfast location showed that significantly more energy was observed eaten for breakfast in the classroom (276 kilocalories) than in the cafeteria (250 kilocalories; p = 0.017). However, a post-hoc two sample *t*-test of observed energy intake for lunch by breakfast location showed no significant difference (p = 0.875; 515 kilocalories in the classroom; 512 kilocalories in the cafeteria).

A post-hoc two sample *t*-test of observed energy intake for breakfast by sex showed that significantly more energy was observed eaten by boys (286 kilocalories) than girls (243 kilocalories; p < 0.001). Likewise, a post-hoc two sample *t*-test of observed energy intake for lunch by sex showed that significantly more energy was observed eaten by boys (540 kilocalories) than girls (487 kilocalories; p = 0.005).

## Discussion

Results from the current analyses showed that daily participation in school breakfast, school lunch, and both school meals combined was not significantly associated with BMI or BMI category (for RQ1 and for RQ3). Although these results agree with those of the Expert Panel convened in March, 2004 [9], the results concerning participation in school breakfast and BMI differ with results from the third School Nutrition Dietary Assessment Study [18] which showed a negative relationship between school breakfast participation and BMI percentile. For that study, children reported their usual weekly participation in school breakfast and in school lunch. However, the current analyses used daily SBP and NSLP participation obtained from electronic administrative records provided by the school district.

Breakfast location was significant for BMI for RQ1, RQ2, and RQ3, and for BMI category for RQ3. When breakfast location was considered in post-hoc analyses, significantly more energy was observed eaten for breakfast in the classroom; however, observed energy intake for lunch by breakfast location showed no significant difference.

The significant effects of sex and age on BMI found in RQ1 were absent in the other two RQs where either observed energy intake or both daily participation and observed energy intake were included in the models. When sex was considered in post-hoc analyses, significantly more energy was observed eaten for breakfast and for lunch by boys.

Observed energy intake at breakfast and observed energy intake at lunch were positively associated with BMI and BMI category for RQ2 and RQ3 with the exception of BMI for observed energy intake at breakfast for RQ2. Similar to results from the current analyses, results from a small study with fourth-grade children showed that observed energy intake at school meals was positively associated with children's BMI percentile [38]. For that study, each of 20 children (15 Black; half girls) of low-BMI percentile (≥ 5^th ^and < 50^th ^percentiles) and 20 children (15 black; half girls) of high-BMI percentile (≥ 85^th ^percentile) was observed eating two school meals (breakfast, lunch) on a school day. More kilocalories were observed eaten by children of high-BMI percentile than low-BMI percentile (p < 0.02), but no significant difference was found in kilocalories reported eaten by children of high-BMI percentile and low-BMI percentile [38].

The current analyses utilized SBP and NSLP participation regardless of whether children received school meals for free, reduced price, or full price. This is appropriate because what is important is SBP and NSLP participation (i.e., obtaining the meal [s] at school) rather than meal price [17,39].

Limitations of the data analyzed include that the validation study that provided data for the current analyses was not designed to address the three RQs, nor was it designed to determine whether higher BMI causes children to eat more, whether eating more causes children to have higher BMIs, or both. No measures of physical activity or observed 24-hour intake were available, and weight/height measurements were available for only one time point per child. The sample included only fourth-grade, primarily Black children from one school district. The estimates of energy yielded by the use of standardized school meal portions may be somewhat imprecise, but the same process was used consistently for each observed child for each observed school meal irrespective of children's BMI or BMI category.

An important strength of the analyses in the current article is that participation in school meals was determined using electronic administrative records of *daily *participation obtained from the school district, rather than crude summary measures of parental- or self-reports of school meal participation (e.g., yes/no dichotomous reports for the year; or weekly summary measures of usual participation). Another strength is that direct observation of school meals was used to determine energy intake; thus, the use of parent's and/or children's reports of children's intake was avoided. An additional strength is that rigorous quality control measures were implemented for school meal observations as well as for weight and height measurements. A final strength is that for all children, weight and height were measured after breakfast, but before lunch.

## Conclusions

The secondary analyses of the current article were a cost-efficient step to add new knowledge and inform the design of future controlled trials and/or cohort studies aimed at understanding pathways to obesity among children who participate in school meals. Longitudinal studies with weight/height measurements at the beginning and end of each school year during elementary school, along with children's daily participation in school meals during elementary school, could help resolve concerns about a relationship between participation in school meals and childhood obesity. School meal observations conducted during each year of elementary school, and other desirable data (such as physical activity), should be included if allowed by budgets.

Additional studies are needed to extend the current article's results which indicated positive relationships between children's BMI and observed energy intake at school meals, and between children's BMI and school breakfast in the classroom. For example, does the energy content of school breakfast differ by location (classroom; cafeteria)? Does the energy content of school meals differ by children's BMI whether offer-vs-serve foodservice is implemented? Results from such studies could provide important guidance for policy changes concerning school meals.

## List of abbreviations

BMI: body mass index; SBP: School Breakfast Program; NSLP: National School Lunch Program; RQ: research question.

## Competing interests

The authors declare that they have no competing interests.

## Authors' contributions

SDB conceived the study, participated in the study's design and coordination, and drafted the manuscript. JWH performed the statistical analyses and helped to draft the manuscript. CHG participated in quality control and data coding, and helped to draft the manuscript. JAR was responsible for data management/programming and helped with statistical analyses. AJM and CMD participated in the recruitment of schools and participants, acquisition of data, quality control, data entry, and data coding. All authors read and approved the final manuscript.

## Authors' information

SDB is a Research Professor in the Institute for Families in Society at the University of South Carolina in Columbia, South Carolina, USA; she is a Registered and Licensed Dietitian, and a Fellow of the American Dietetic Association. JWH is a Research Associate Professor in the Department of Epidemiology and Biostatistics, and Director of the Biostatistics Collaborative Unit at the University of South Carolina in Columbia, South Carolina, USA. CHG is a Research Dietitian in the Institute for Families in Society at the University of South Carolina in Columbia, South Carolina, USA; she is a Registered and Licensed Dietitian. JAR is a Research Associate in the Institute for Families in Society at the University of South Carolina in Columbia, South Carolina, USA. AJM is a Research Specialist II in the Institute for Families in Society at the University of South Carolina in Columbia, South Carolina, USA. CMD is a Research Dietitian in the Institute for Families in Society at the University of South Carolina in Columbia, South Carolina, USA; she is a Registered and Licensed Dietitian.
